# Fully Polymeric Distillation Unit Based on Polypropylene Hollow Fibers

**DOI:** 10.3390/polym13071031

**Published:** 2021-03-26

**Authors:** Tereza Kůdelová, Erik Bartuli, Alan Strunga, Jiří Hvožďa, Miroslav Dohnal

**Affiliations:** 1 Heat Transfer and Fluid Flow Laboratory, Faculty of Mechanical Engineering, Brno University of Technology, Technická 2, 61669 Brno, Czech Republic; Erik.Bartuli1@vut.cz (E.B.); Alan.Strunga@vut.cz (A.S.); Jiri.Hvozda@vut.cz (J.H.); 2 ZENA s.r.o., Branky 278/21, 66449 Ostopovice, Czech Republic; dohnalml@zena-membranes.cz

**Keywords:** polypropylene, hollow fiber membranes, heat transfer, sweep gas membrane distillation

## Abstract

Access to pure water is a very topical issue today. Desalination represents a promising way of obtaining drinking water in areas of shortage. Currently, efforts are being made to replace the metal components of existing desalination units due to the high corrosivity of sea water. Another requirement is easy transportation and assembly. The presented solution combines two types of polymeric hollow fibers that are used to create the distillation unit. Porous polypropylene hollow fiber membranes have been used as an active surface for mass transfer in the distillation unit, while non-porous thermal polypropylene hollow fibers have been employed in the condenser. The large active area to volume ratio of the hollow fiber module improves the efficiency of both units. Hot water is pumped inside the membranes in the distillation unit. Evaporation is first observed at a temperature gradient of 10 °C. The water vapor flows through the tunnel to the condenser where cold water runs inside the fibers. The temperature gradient causes condensation of the vapor, and the condensate is collected. The article presents data for hot water at temperatures of 55, 60, and 65 °C. Optimization of the membrane module is evaluated and presented.

## 1. Introduction

Membrane distillation is one of the newer separation processes, in which the individual components are separated based on their different molecular properties. The process utilizes mass transfer between the gas and liquid phase. A membrane has defined surface properties as well as a precisely defined pore size through which the gas phase of the substance penetrates. Theoretically, the separation yields a 100% pure compound, cleansed of all salts or contamination [1,2,3]. Phase transition occurs in membrane distillation, therefore higher energy requirements should be assumed than for processes without it [1].

This process has a wide range of potential applications including desalination, wastewater treatment, heavy metal removal, and processes in the food industry, although it has not yet been implemented industrially. Most current applications of membrane distillation are still in the laboratory or small pilot stage. Thus far, the primary focus has been water desalination. The possibility of using renewable energy sources, such as waste heat, solar energy, or geothermal energy, may allow the integration of membrane distillation with other processes, making it more favorable energy-wise and therefore promising on an industrial scale [4,5]. Another possible use of the polymeric hollow fiber membrane is for aerosol or gas filtration [6,7,8,9].

Microchemical process technologies have been identified as a suitable strategy for intensification of chemical processes [4]. In these technologies, the modules have at least one dimension less than 1 mm. The streamlining of microchemical processes is possible mainly through the fact that when the liquid flows inside a channel which has a diameter of less than 1 mm, its heating or cooling is more efficient due to a significant increase in the ratio of the active area to the module volume [10]. By reducing the diameter of the channels used, the distance of the liquid from the active membrane surface is also reduced.

Mass transfer in membrane distillation is controlled by three basic mechanisms: Knudsen diffusion [11], Poiseuille flux (viscous flux) [12], and molecular diffusion [13]. The Knudsen number, defined as the ratio of the mean free path of transported molecules to the membrane pore size, is an indication of which mechanism is predominant within the membrane pores. The prevailing mechanism defines the type of mass transfer resistance due to a momentum transfer to a supported membrane (viscous flux resistance), collision of molecules with other molecules (molecular resistance), or collision with the membrane itself (Knudsen resistance). The resistance in the boundary layer is generally negligible, as is surface resistance, because the surface area is small compared to the pore area. On the other hand, the thermal boundary layer has been shown to be a limiting step to mass transfer [14].

Over a billion people do not have access to clean drinking water [15]. Even though existing drinking water supplies are sufficient for the general population, the distribution of drinking water does not coincide with population distribution [16,17]. Membrane distillation looks like a promising technology to solve this pressing issue, and therefore a lot of progress has been made in this field.

Generally, membrane distillation works at lower temperatures than other conventional distillation methods and runs at lower hydrostatic pressures than other membrane-based processes, making it a more economically advantageous operation, especially since it also has less demanding membrane mechanical properties [4].

The membranes used in membrane distillation can be configured into various membrane modules, the most common being plate-and-frame, hollow fibers, tubular, and spiral-wound. The main choice of membrane module depends on the operating conditions and costs. Other important performance criteria include effective control of the temperature and concentration effects. Plate-and-frame type configurations are widely used in the research for this paper because they are easy to clean and replace. However, their high price and low surface area to volume ratio makes them uneconomical for the industry. Hollow fiber or spiral-wound modules are usually used instead [4,14,18,19].

Membranes are defined by several parameters, the most important being liquid entry pressure (LEP), hydrophobicity/hydrophilicity, permeability, chemical and thermal stability, and fouling rate.

Liquid entry pressure (LEP) is the minimum hydrostatic pressure that must be applied to the solution to overcome the hydrophobic forces, and undesirable leakage will occur. It can be calculated as follows:

(1) $$\mathrm{LEP}=\frac{B\gamma_{L}cos\varphi }{r_{max}}$$

where *B* is the geometrical factor that is defined by the properties of the pores, γ*_L_* is the surface tension of the liquid, *φ* is the contact angle of the liquid with the solid surface, and *r_max_* is the maximum pore size [20].

Wettability of the membrane is an important parameter of the membrane, which influences the LEP. Wettability can be defined by the contact angle, which is the angle formed by the membrane surface by the tangent to a drop of the liquid [21]. Hydrophilic materials have a contact angle lower than 90°, i.e., the liquid spreads on the surface easily. A contact angle higher than 90° characterizes hydrophobic materials. The dynamic contact angles measured by the Wilhelmy balance method for non-porous polymeric hollow fibers were studied before [22]. A contact angle of above 90° is required for applications with saline aqueous solutions.

The permeability of the membrane affects the amount of the output—the higher the permeability, the higher amount of the output. Molar flux depends on the porosity *ε* of the membrane, the tortuosity τ of the pores, membrane thickness δ and the average pore size ⟨*r*^α^⟩, and is defined as [23]:

(2) $$N\propto \frac{\langle r^{\alpha }\rangle \varepsilon }{\tau \delta }$$

Membrane porosity is another important parameter of the membrane that influences the permeability. A membrane with higher porosity provides more active surface area, which contributes to the higher mass transfer through the membrane and decreases the thermal conductive losses through the membrane wall [24].

To ensure long-term stability and functioning of the modules, the membrane must have high chemical stability to avoid any reaction between the solution and the membrane that could cause damage to the membrane structure. Moreover, thermal and mechanical stability must be ensured to avoid degradation of the membrane during operation.

The membrane thickness significantly influences the process of penetration through the membrane. The lower thickness contributes to the higher mass transfer through the membrane. However, it also contributes to higher thermal losses that negatively affect the energy requirements [25]. This has led to the use of composite membranes that have more layers [26]. Composite membranes have a thin hydrophobic selective layer and a hydrophilic supporting layer.

The size of the pores determines the mass transfer and LEP. Larger pores have higher mass transfer, but also lower LEP [24]. Therefore, it is necessary to optimize the pore size. Commonly used membranes have a pore size in the range of 100 nm–1 µm [13]. The membranes used in this study have an average pore size of 0.1 µm and were produced by ZENA s.r.o. [27].

The phenomenon of membrane fouling is one of the most serious issues of membrane distillation and hollow fiber membranes with a small diameter are especially vulnerable. In order to prevent it, filtration of the solution from impurities must be ensured [28]. Membranes are usually fouled by various salts, predominantly compounds of calcium and magnesium [29], that form scales in the area of the pores [30]. Once the initial fouling layer is formed it starts to grow rapidly. The thickness of the fouling layer affects both the mass and heat transfer [18,31]. The formation of scales is referred to as the main reason for undesirable leakage, decrease in the mass transfer, and damage to the membrane structure [32]. Increased temperature propagates the formation of scales [33]. It is necessary to eliminate fouling as much as possible to guarantee reliable operation of the membrane distillation unit. A gas added to the solution is one of the possible ways to reduce fouling [34]. Another possibility is to use polyphosphate, which does not inhibit the formation of the scales, but prevents them attaching themselves to the membrane wall [35]. In addition, modification of the membrane wall surface can prevent scales from adsorbing [36].

Non-porous polypropylene hollow fibers were used in the design of polymeric hollow fiber heat exchangers (PHFHEs). The first mention of PHFHEs is from 2004 in [37], where the authors used PHFHEs as condensers. PHFHEs consist of many polymeric hollow fibers (hundreds or even thousands) with an outer diameter of around 1 mm. The thickness of the wall ranges from 10% to 25% of the fiber outer diameter depending on the application requirements. PHFHEs are an alternative to the commonly used metal heat exchangers and have several advantages including lower weight, lower material costs, corrosion resistance, easier production and modification, and high chemical endurance and stability. Moreover, PHFHE also require less energy to produce and are recyclable, which contributes to their environmental friendliness [38]. Polymers are known for their low thermal conductivity, which ranges from 0.1 W/(m K) to 0.4 W/(m K), which could be their main disadvantage. However, it can be neglected due to the very low thickness of the fiber wall.

PHFHEs are very compact heat exchangers that provide a large heat transfer area with respect to their volume [39]. PHFHEs can have a chaotic, semi-chaotic, or regular structure [40]. Chaotization of the polymeric hollow fibers contributes to the improved use of the active heat transfer area of the unit. Separation of the fibers is necessary to guarantee high efficiency [41]. The importance of the fiber arrangement is highlighted in [42,43], where the authors claim that tilted fibers forming an angle of 22.5° across the layers, which results in an overall increase of 12.5% in the heat transfer coefficient in comparison to the parallel fibers.

Possible fouling of PHFHEs must be taken into consideration. The fouling can occur on both the inner and outer surfaces of hollow fibers. Internal fouling is dealt with in the same way as for membrane fibers, i.e., filtration of mechanical impurities and use of polyphosphate [35]. There are two types of fouling of PHFHEs on the outer surface: Organic and inorganic. In the case of organic fouling, once the initial fouling layer is formed, fouling grows rapidly [44]. PHFHEs show better results for inorganic fouling than aluminum heat exchangers that are commonly used in air-conditioning [45]. A PHFHE with a regular structure was used in the study.

Determination of the lifetime is an important factor in every device evaluation. Fatigue testing by pressure loading proved that chaotic PHFHEs are able to withstand more than one million pressure cycles (from 0 to 3.5 bar) without any sign of damage [46]. These PHFHEs are used in this study. PHFHEs can be used as immersed heat exchangers that are competitive in comparison to commercially available heat exchangers. They have overall heat transfer coefficients of up to 890 W/(m^2^ K) [47]. Another possible use is in the automotive industry as a car radiator. The study [48] concluded that PHFHEs are able to achieve comparable results to metal finned tube heat exchangers.

PHFHEs are also suitable for HVAC applications [45]. Condensation on the outer surface of PHFHEs is influenced by the wettability of the fibers. The characterization of the outer surface wettability of the polypropylene hollow fibers that are used for production of PHFHEs is presented in [22]. The hydrophobic polypropylene causes dropwise condensation [49,50]. The possibility of using PHFHEs for cooling electronic systems is described in [51]. Due to their flexibility, PHFHEs can be used for cooling electronic boxes that can be difficult to access. The study shows that the overall heat transfer coefficients in water-air application are up to 250 W/(m^2^K) and 80 W/(m^2^K) for forced and natural convection, respectively.

PHFHE as a super compact cooler for Li-ion cells is presented in [52]. A housing made of polydicyclopentadiene is used for polymeric hollow fibers to create a fully polymeric solution. The flexibility of the fibers allows the fibers to be wound around the Li-ion cell and hence to achieve a large contact area and effective cooling. In the study presented in this paper, the polypropylene PHFHE with a chaotic structure will be used as a condenser.

This study examines the combination of the polymeric hollow fiber membrane which can be used for membrane distillation, and PHFHEs that have already proved their endurance for pressure loading and showed large overall heat transfer coefficients. Despite the fact that they are made of polypropylene, they are an effective heat exchanger with resulting benefits such as low weight, low energy consumption during manufacturing, and chemical and corrosion endurance.

## 2. Materials and Methods

### 2.1. Methods

Depending on how the vapor pressure gradient is induced, membrane distillation can be divided into four types: Direct contact membrane distillation (DCMD), vacuum membrane distillation (VMD), air gap membrane distillation (AGMD), and sweep gas membrane distillation (SGMD).

DCMD is a very simple configuration in which the membrane separates two media at different temperatures. The temperature difference between the two media induces a vapor pressure difference across the membrane. This difference is responsible for the evaporation of the warm solution molecules. Vapor is then driven by the force caused by the pressure difference through the pores of the membrane and permeates it. When the vapor comes into direct contact with the permeate, it condenses due to the lower temperature and pressure [4,53]. The warm solution is maintained at atmospheric pressure and below its boiling point. The colder permeate is maintained at a significantly lower temperature and is circulated under the same conditions as the warm solution. The hydrostatic pressure must not exceed the maximum permissible value on either side of the membrane, otherwise undesirable leakage will occur.

AGMD is a variant of membrane distillation in which there is a stationary air gap between the membrane and the surface on which the vapor condenses. This surface is built into the module. Due to the thermal characteristics of the air (thermal insulator), the air gap contributes to the reduction of heat loss due to conduction. The disadvantage of the air gap is the additional convection resistance, which must be overcome when vapors penetrate the membrane. This negatively affects the mass flow through the membrane [4,53].

VMD is based on the use of low pressure or a vacuum on the permeate side. The pressure is lower than the saturation pressure of the volatile molecules that will be separated from the solution. The vapor condenses outside of the membrane module. The advantages of this configuration are the low conduction heat losses and reduced mass transfer resistance. On the other hand, its disadvantages are the increased risk of pore wetting and higher energy requirements [4,53].

SGMD uses cold sweep gas to transport the vapor to the condenser. The mechanism is similar to AGMD, however the sweep gas reduces the convection resistance and improves the mass transfer. Usually, air or an inert gas like nitrogen is used in SGMD as the sweep gas. The condenser is placed outside the membrane module. Diffusion of a small volume of vapor into a large volume of swept gas is the disadvantage of SGMD [4,53].

DCMD is the most studied type of membrane distillation due to its simplicity [54]. The advantage of VMD is the high product yield. The advantage of AGMD and SGMD is the ratio between output and energy costs [55,56,57]. AGMD is also considered to be the most flexible configuration and therefore has potential in desalination [58].

SGMD was chosen as the type of membrane distillation for the research presented in this paper. Air was used as the sweep gas.

### 2.2. Experimental Part

**Figure 1:**
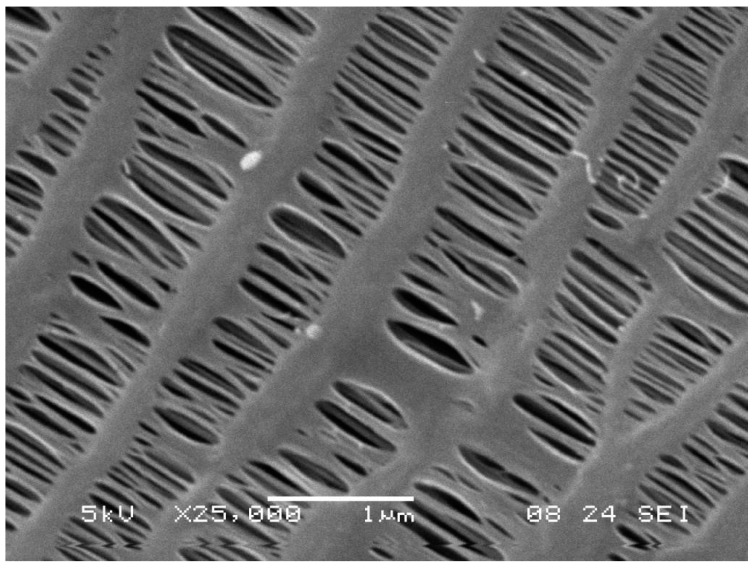
An electron microscope image of the porous polypropylene membrane that was used for distillation; the photograph was provided by ZENA s.r.o. [27].

**Figure 2:**
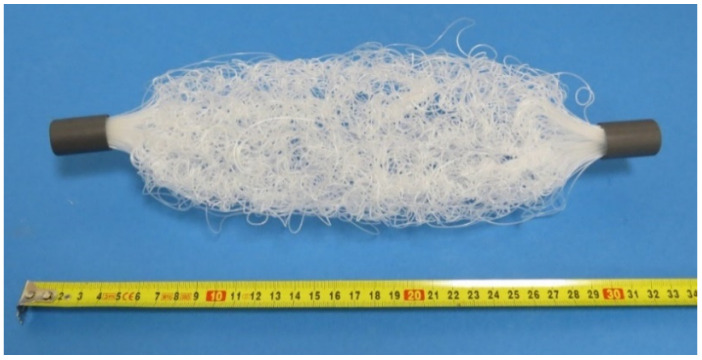
A condenser made of non-porous polypropylene hollow fibers.

The distillation unit consists of three main parts—a distillation tunnel, a membrane module, and a condenser. The tunnel is made of transparent polycarbonate sheets so the process can be continuously observed. The membrane module consists of a bundle or a number of bundles of polymeric hollow fiber membranes. The membranes used were produced by ZENA s.r.o. [27]. An electron microscope photograph of the membrane is shown in Figure 1. These membranes are made of hydrophobic polypropylene fibers with an outer diameter (OD) of 0.6 mm, an inner diameter (ID) of 0.48 mm, membrane thickness of 0.06 mm, average pore size of 0.1 µm with a porosity of 50%, and LEP of over 3.5 bar. The condenser is made of polypropylene hollow fibers as well, however, these are non-porous. The OD of the condenser’s fibers is 0.8 mm and the ID is 0.6 mm. The condenser is made of 200 fibers with a length of 600 mm. Therefore, the condenser provides a heat transfer area of 0.3 m^2^. The condenser is shown in Figure 2.

**Figure 3:**
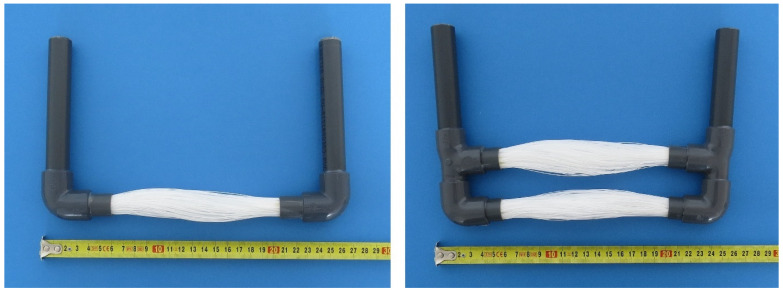
Single-bundle membrane module MM003 (**left**) and double-bundle membrane module MM006 (**right**).

The optimization of the membrane module required six different configurations. They differ in the number of fibers, the OD of the fibers, and the length of the fibers. All bundles were potted in a DN 16 PVC tube. The properties of the membrane modules are listed in Table 1. Examples of a single-bundle and double-bundle membrane module are shown in Figure 3.

The test tunnel has a rectangular cross-section with a height of 120 mm and a width of 100 mm. The tunnel forms a rectangular 2000 mm × 1000 mm closed loop. The test section is located along the longer side. On the other side of the tunnel is the spot where the temperature, humidity, and air speed are measured in stabilized conditions. A multiple of 10 hydraulic diameters is applied in front of and behind the test spot.

The membrane module and the condenser are placed into the distillation tunnel where the sweeping air flows. The air flow is provided by a standard computer fan with dimensions of 100 mm × 100 mm. The fan was supplied with 11 V for each configuration, which corresponds to an air speed from 0.6 to 0.8 m/s relative to the specific module. The hot medium flows through the distillation membrane module. The water evaporates and the water vapor penetrates the membrane in the tunnel. It is then swept along by the flowing air to the condenser, where it condenses and the condensate is collected. Tap water was used as a hot medium for verification of the distillation unit. Before the experiment, the water was modified with polyphosphate. This treatment is based on the ability of phosphate polymers to be absorbed by the surface of crystalline calcium and magnesium cores. This creates a protective film that prevents the cores from merging and forming crystals. Water treated this way, while maintaining its original hardness, does not form a harmful coating. Drinking water remains drinkable even after this treatment [59]. The concentration of the employed solution was 0.02 g/L.

**Figure 4:**
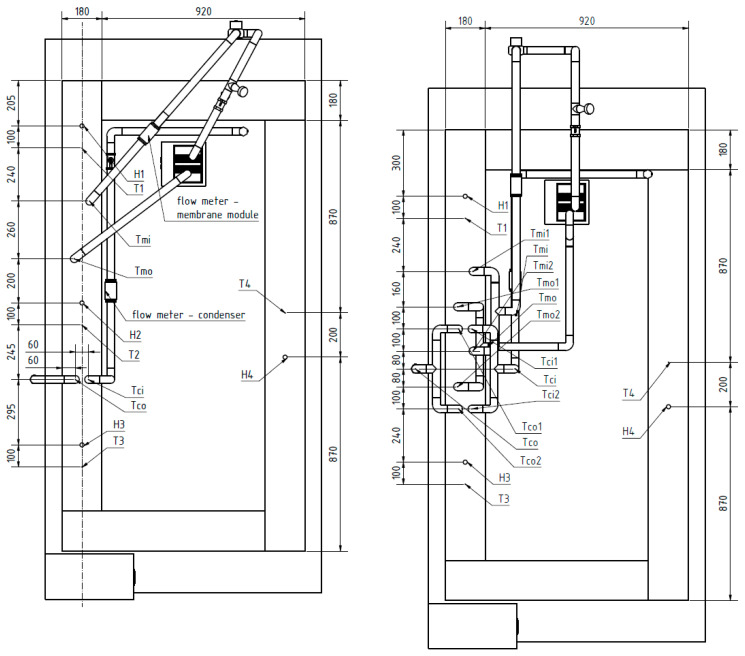
Scheme of the test rig for MM001 (**left**) and for other membrane modules (**right**), top view.

The scheme of the test rig with the positions of the measuring probes is shown in Figure 4. The input and output temperature, pressure drops, and flowrate are measured for the membrane module and the condenser. The humidity and temperature are measured inside the tunnel. The measuring spots are before and after the tested section, and one spot is opposite the testing section where the air flow is stabilized. This is also the spot where the air speed is measured. All temperatures are measured with the Pt100 thermometer (OMEGA Engineering, Inc., Norwalk, CT, USA) with accuracy of 1/3 DIN, i.e., ± (0.10 + 0.0017·abs (t)) °C. The humidity meter (B+B Thermo-Technik GmbH, Donaueschingen, Germany) gives an error of ± 3% of the measured value relative humidity, the anemometer (OMEGA Engineering, Inc., Norwalk, CT, USA) has an accuracy of ± (5% of measured value + 0.1) m/s, and the pressures (KELLER AG für Druckmesstechnik, Winterthur, Switzerland) are measured with an error of ± 25 Pa. The hot medium flowrate (ifm electronic, Essen, Germany) has an error of 0.4 L/min and the flowrate through the condenser has an accuracy of ±0.8 L/min. The relative humidity and temperature measured inside the tunnel are marked as H1, …, H4 and T1, …, T4. The air inside the tunnel flows from H1 to H4 through H2 and H3. As mentioned above, Tmi marks the input temperature of the membrane module and Tmo is the output temperature of the membrane module. Tci denotes the input temperature of the condenser and Tco denotes the output temperature of the condenser. The pressure at the input and output of the membrane module and condenser were measured at the same spot as temperatures Tmi, Tmo, Tci, and Tco. When testing two membrane modules simultaneously, index numbers 1 and 2 are added to the temperatures. These denote the first or second membrane module or condenser in the direction of the air flow. H2 and T2 were not measured when the scheme on the right side of Figure 4 was applied. The notations H3, H4, T3, and T4 remain the same as in the scheme on the left for reasons of coherence. See Figure 4 for detailed locations of all measuring spots.

The influence of the input temperature of the membrane module was expected. 55 °C, 60 °C, and 65 °C were chosen as testing temperatures. The humidity and temperature of the air were measured to observe the capability of the air to absorb and release the moisture. 

The Knudsen number (Kn) was evaluated for the testing temperatures 55 °C, 60 °C, and 65 °C; its values are 3.36, 2.67, and 2.19, respectively. The transition regime occurs for 1 < Kn < 10. The mean free path of a molecule is comparable to the average pore size and the diffusion is a mixture of the free and the confined modes of diffusion.

**Figure 5:**
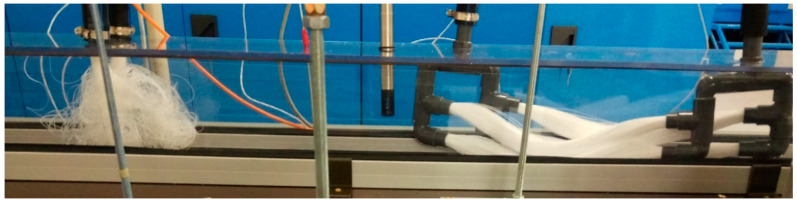
MM001 (**right**) with the condenser (**left**) inside the tunnel, the air flows from the right to the left.

Firstly, the membrane module MM001 was used. The membrane module consists of four bundles, each with 1000 fibers with an OD of 0.33 mm. MM001 is the module with the largest active surface—1.17 m^2^. Therefore, the largest amount of condensate was expected here. Due to the length of the fibers and their small OD, the fibers were too flexible, and they thus slipped into four big tubes instead of 4000 small ones, and the active surface was blocked by the contact with each other. Therefore, the permeate flux of the module was poor. Figure 5 shows the MM001 and the condenser inside the tunnel.

**Figure 6:**
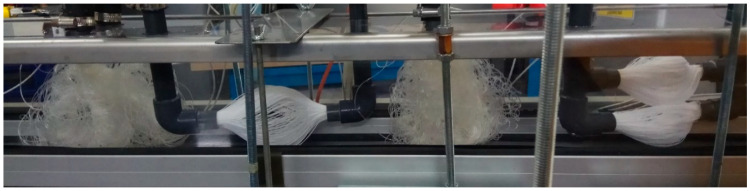
Testing section with MM007, condenser, MM004 and condenser (from right to left).

Based on the measured results of MM001, the other membrane modules are made of 140 mm fibers with an OD of 0.6 mm. These modules provide a much lower mass transfer area, but the fibers are stiffer. The length of 140 mm was chosen as it is the longest fiber option that can still be separated. The separation is done by pushing the module terminals towards each other by 20% of the original length. This method does not affect the hydrophobicity of the fibers and causes no kinks. The separation of the fibers is shown in Figure 6. It can be also observed that when the module is made of two bundles, the separation of fibers is worse. This can in particular be seen in Figure 6, where the bottom bundle is limited by the separation of the fibers in the upper bundle. Although the separation of the bundles is similar (due to the dimensions of the fibers and their number), it is never identical. This can result in a slightly different mass transfer area. However, the difference is not significant as the membrane fibers are randomly touching each other.

## 3. Results

The results are presented separately for individual membrane modules. The lower index m, c, i, o marks the membrane module, condenser, input, and output, respectively. Each experiment is given a reference-EXxy, where xy is a number from 01 to 20 for better orientation.

### 3.1. Four-Bundle Membrane Module

The test scheme for measuring MM001 is shown in Figure 4 on the left. MM001 was tested at two different membrane flowrates, (Qm) 100 and 60 L/h, and for three different input temperatures in the membrane module, (Tmi) 55, 60, 65 °C. The flowrate through the condenser was 360 L/h for all measurements. The air speed inside the tunnel was 0.7 m/s. The measured values can be seen in Table 2 and Table 3. Error states the absolute value of the measurement error that comes from the thermal balance between the membrane module and the condenser. The air temperature in the tunnel was 19 °C in the spot where the air speed was measured.

Optimization of the membrane module was carried out based on the results of MM001. The OD and ID are larger, and fibers are shorter for the other membrane modules.

### 3.2. Membrane Modules with Bundles Consisting of 500 Fibers

MM004 and MM007 are made of bundles of 500 fibers to achieve the largest possible mass transfer area with respect to the OD of the membranes and the ID of the PVC tube used for the potting. Two condensers were used in combination with MM004 and MM007. One condenser was placed behind each membrane module, see Figure 6. The scheme is shown in Figure 4 on the right.

The total flowrate through both condensers was constant at 550 L/h. The air speed was 0.6 m/s. The measured values are shown in Table 4 and Table 5.

### 3.3. Membrane Modules with Bundles Consisting of 300 Fibers

Membrane modules MM003 and MM006 were tested separately in combination with one condenser. The scheme is shown in Figure 4 on the right. The membrane module and the condenser were placed at the point marked 1. The flowrate through the condenser was 360 L/h. The air speed was 0.7 m/s. The measured values are listed in Table 6 and Table 7 for MM003, and in Table 8 and Table 9 for MM006.

### 3.4. Membrane Module with Bundle Consisting of 200 Fibers

**Figure 7:**
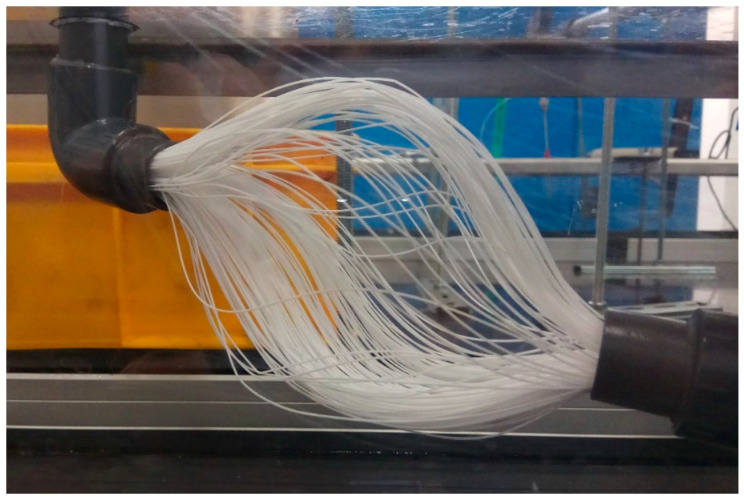
MM002 during experiment.

Only one membrane module with a 200 fibers bundle was tested. MM002 was measured in combination with one condenser. The scheme is shown in Figure 4 on the right. The flowrate through the condenser was 360 L/h. The air speed was 0.8 m/s. The separated fibers of MM002 during the experiments are shown in Figure 7. A single-bundle membrane module provides the possibility of separation by varying the height of the bundle terminals. The measured values are in Table 10 and Table 11.

## 4. Discussion

The limescale, which is formed when hard water is used, is able to completely block the pores and the membranes inside. It is impossible to avoid the use of hard water when the distillation unit is used for desalination. The hardness of the tap water used was 3.02 mmol/L. Therefore, the polyphosphate was added to the water to prevent the crystalline calcium and magnesium cores from merging and forming crystals. The approach of polyphosphate water treatment during desalination is described in [35]. Drinking water remains drinkable even after this treatment [59].

Testing of MM001 has shown that using too many very long thin fibers is not a promising approach. Specifically, 4000 fibers of 300 mm length with an OD of 0.33 mm were used. These fibers were too soft to separate. The fibers kept the shape of four bundles, each of 1000 fibers. Therefore, the mass transfer area of 1.24 m^2^ was not efficiently used. The four bundles have a surface area of 0.06 m^2^ when made from one large tube with the same external dimensions as the bundles used. That is 20 times less. Manipulation of the membrane module was rather difficult because the hydrophobic fibers cannot be touched so as to preserve their functionality. The long and very narrow fibers were not tough enough to remain in position when the terminals of bundles were pushed towards each other and the fibers were partially separated. Hence, the module showed insufficient permeate flux in comparison with other membrane modules, and a maximum permeate flux of 0.79 kg/m^2^h was achieved. The influence of the flowrate is not significant, as an increase in the flowrate of 66% resulted in an increase in the permeate flux of 2.5–7.5% depending on the temperature.

In the next step, fibers with an OD of 0.6 mm were used for other membrane modules to obtain tougher fibers that are still small enough to reach a large mass transfer area. The length of 140 mm was chosen as the maximum possible length of fibers that are easily separable by pushing the terminals towards each other. Bundles made of 200, 300, or 500 fibers were used.

Firstly, a combination of the single and double-bundle modules of 500 fibers was used to provide as large a mass transfer area as possible. The membrane modules made of bundles with 500 fibers were tested together, i.e., the single and double-bundle modules were combined with two condensers. The fibers were able to separate, as can be seen in Figure 6. An increase of 30% in permeate flux was observed at a temperature of 55 °C when the membrane flowrate was increased by 50%. By increasing the membrane flowrate by 25% at a temperature of 60 °C the permeate flux was increased by 18%. When the temperature inside the membrane module was increased from 55 °C to 60 °C the permeate flux increased by 8%. The increase of 15% in the permeate flux was observed when the temperature was increased from 60 °C to 65 °C, and at the same time, the membrane flowrate was increased by 140%. This resulted in an increase of 320% in the membrane pressure drop. The best way to operate the combination of MM004 and MM007 is at a membrane flowrate of 150 L/h and a temperature of 60 °C due to the high permeate flux and low pressure drop, i.e., when the energy requirements are lower.

The previous testing of the modules with bundles of 500 fibers showed that the air inside the tunnel was too wet. Therefore, efforts to decrease the mass transfer area in order to increase the absorption of the humidity of the air were made. Hence, the number of fibers was reduced and the membrane modules made of 300 fiber bundles were tested individually, i.e., the single-bundle module was tested in combination with one condenser as well as the double-bundle module. The increase in temperature from 55 °C to 65 °C led to an increase of 36% in the permeate flux at the same pressure drop. Double-bundle module MM006 showed much lower permeate flux than single-bundle module MM003. The permeate flux of MM006 was 42% lower at 65 °C even though the membrane flowrate was 66% higher for MM006. The increase in temperature from 60 °C to 65 °C resulted in a 16% increase in the permeate flux. However, the pressure drop of MM006 is significantly lower, as can be expected. The total amount of condensate in mL/h is higher for MM006 at a lower pressure drop. Hence, MM006 seems to be more promising than MM003.

The number of fibers was decreased to 200 to observe the influence of better separation of the fibers. As can be observed from Figure 7, the fibers are very well separated when the bundle of 200 fibers is used. The permeate flux of the membrane module is high as well. However, the total amount of condensate is low (130 mL/h), and the pressure drop is high. The amount of condensate does not depend on the temperature for MM002, as the amount is always 130 mL/h. This is due to the effective use of the mass transfer area of the membrane module.

For the fibers with a length of 140 mm, the highest amount of condensate (300 mL/h) was obtained for a combination of MM004 and MM007 in experiment EX11. However, the permeate flux was only 0.75 kg/(m^2^h), which is the lowest of all the membrane modules. The highest permeate flux of 2.61 kg/(m^2^h) was obtained in experiments EX18, EX19, and EX20 when MM002 was used. On the other hand, the amount of condensate was only 130 mL/h, which is the lowest from all the experiments. As mentioned, the fibers were perfectly separated and effectively used, but the mass transfer area was too small to provide a larger amount of condensate. This also resulted in a large pressure drop that affected the consumption of energy during its operation. A 38% higher amount of condensate was obtained in EX15 with a pressure drop just 35% of that for MM002. The permeate flux of MM006 in EX15 was 1.13 kg/(m^2^h).

**Figure 8:**
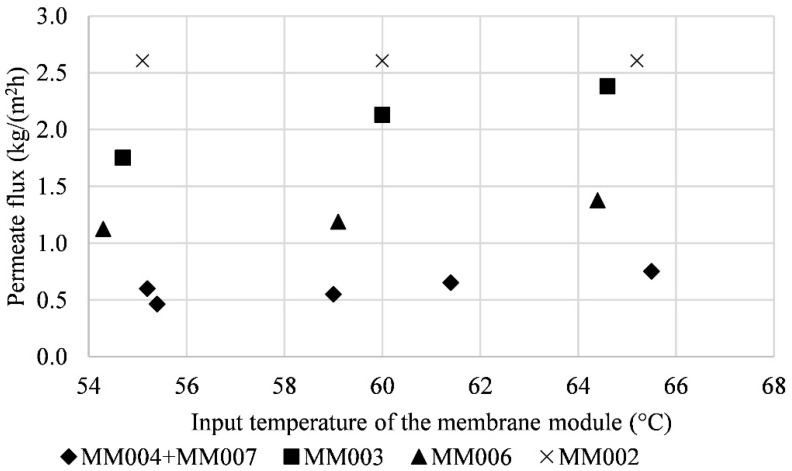
The comparison of the permeate flux depending on the input temperature of the membrane module.

**Figure 9:**
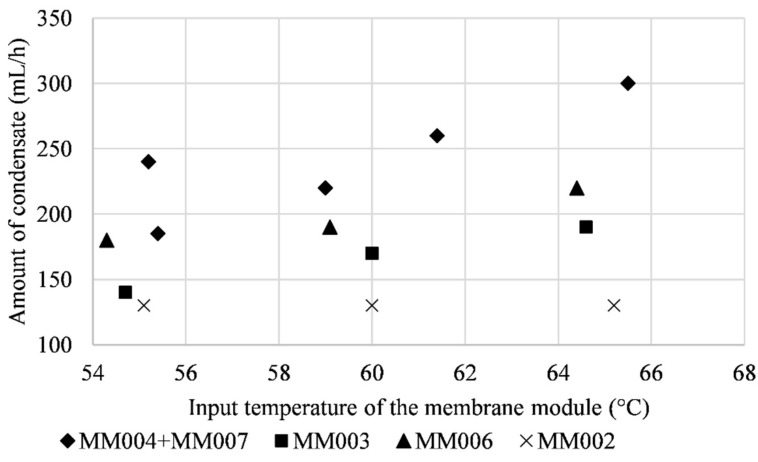
The dependence of the amount of condensate on the input temperature of the membrane module.

An influence of the input temperature of the membrane module on the amount of condensate was expected. The temperatures of 55 °C, 60 °C, and 65 °C were chosen for testing. The input temperature did not show a significant impact on the permeate flux. This was probably caused by the high humidity of the air inside the tunnel and its incapability to absorb another vapor. The comparison of the permeate flux depending on the input temperature of the membrane module is shown in Figure 8. Figure 9 shows the dependence of the amount of condensate on the input temperature of the membrane module.

Table 12 shows the comparison of the measured data in EX14, EX17, and EX18 with results published in [60,61,62,63]. Significantly higher flowrates were used in this study. The settings differ in solution used, temperatures, and flow rates. The studies [60,61,62] show the possible application of the tested setup where the membrane distillation was used to separate water and sodium chloride or nitric acid. Comparable permeate flux with EX14, EX18 was reached in [61], where the solution of sodium chloride in membrane distillation was used. Significantly higher results of the permeate flux are reported in [62], where the sodium chloride solution with similar concentration was used at lower input temperature and higher flowrate. The lowest permeate flux (0.5 kg/m^2^h) was achieved in [60], where the solution of nitric acid with much higher density was used. The highest permeate flux (5 kg/m^2^h) was reached in [63]. This study provides the best comparison as the pure water at similar feed input temperature was used for membrane distillation. The study differs significantly in the flow velocity. The permeate flux reported in [63] is double the one measured in EX14 and EX18. Unfortunately, the amount of condensate is not available and cannot be compared. The results indicate that the permeate flux in our setting could be further improved. More emphasis on fiber separation should be placed as well as on the dehumidification of the air inside of the tunnel.

## 5. Conclusions

The paper presents a membrane distillation unit in which the key parts (mass and heat transfer surfaces) are made of hydrophobic polypropylene. This helps to prevent corrosion. The condenser is a polymeric hollow fiber heat exchanger with a chaotic structure. A series of 20 experiments that verify the functionality of distillation membrane unit was introduced. Six different membrane modules were studied. It was observed that as the temperature increases the permeate flux and the amount of condensate increases slightly. The installation of more condensers should help increase the amount of condensate as the air humidity will decrease and absorption of the vapor will increase.

The measurements proved that the use of polyphosphate prevents the fouling of the hollow fibers with limescale. The drinkability of the water is not affected. This helps reduce thermal losses, though they are increased due to the thermal resistance of the fouling layer.

The study showed that long and thin membrane fibers are difficult to separate. Specifically, this problem affected the membrane module consisting of 4 bundles of 1000 fibers 300 mm long. Therefore, the fibers of 140 mm fibers with a diameter of 0.6 mm were used for further testing. These were stiff enough to be easily separated by pushing the module terminals towards each other by 20% of the original module length. The separation of the fibers is never identical for different modules. This difference is not significant as the fibers are randomly touching each other. Membrane modules made of bundles consisting of 500, 300, or 200 fibers were studied. The efforts to achieve the largest possible mass transfer area led to the testing of membrane modules with 3 times 500 fibers. It was observed that the air inside the tunnel had high humidity, which negatively affected its capability to absorb the vapor. Therefore, the two membrane modules were tested separately, one single and one double-bundle module, with bundles made of 300 fibers. The results showed satisfactory yields. However, the permeate flux of 1.38 kg/(m^2^h) shows that the fibers could be better separated. For that reason, the single-bundle module of 200 fibers was tested. The permeate flux of 2.61 kg/(m^2^h) was achieved, but due to the low mass transfer area, a low amount of condensate (130 mL/h) was produced.

The use of more double-bundle membrane modules in which the bundles consist of 300–500 fibers seems to be a promising solution for membrane distillation and for water desalination application. Combining them with polymeric hollow fiber heat exchangers creates a fully polymeric membrane distillation. The use of a larger number of condensers could beneficially influence the permeate flux of the unit by reducing the air humidity. The functionality of this unit for desalination applications should be verified.

## 6. Patents

The main idea of this research is protected by utility model CZ 32427 U1 “A membrane distillation module” issued by the Industrial Property Office of the Czech Republic.

## Figures and Tables

**Table 1:** Properties of membrane modules.

Membrane Module	No. of Fibers	OD (mm)	ID (mm)	Fiber Length (mm)	Mass Transfer Area (m^2^)	Note
MM001	4000	0.33	0.24	300	1.24	four-bundle membrane module
MM002	200	0.6	0.48	140	0.06	single-bundle membrane module
MM003	300	0.6	0.48	140	0.08	single-bundle membrane module
MM004	500	0.6	0.48	140	0.14	single-bundle membrane module
MM006	600	0.6	0.48	140	0.16	double-bundle membrane module
MM007	1000	0.6	0.48	140	0.29	double-bundle membrane module

**Table 2:** Measured temperatures and humidity for MM001.

	Q_m_ (L/h)	T_mi_ (°C)	T_mo_ (°C)	T_ci_ (°C)	T_co_ (°C)	H1 (%)	T1 (°C)	H2 (%)	T2 (°C)	H3 (%)	T3 (°C)	H4 (%)	T4 (°C)
EX01	60	55.1	52.2	10.6	11.1	83.3	19.9	93.2	24.9	88.0	17.3	88.2	19.0
EX02	100	55.1	53.4	10.6	11.1	82.7	19.8	93.4	24.6	89.7	17.4	85.9	19.0
EX03	60	60.1	57.2	10.5	11.0	93.2	19.5	92.7	25.1	89.6	17.7	85.0	18.7
EX04	100	60.2	58.5	10.2	10.7	81.0	19.6	93.3	24.8	85.8	17.3	84.1	18.8
EX05	60	65.0	61.9	10.7	11.2	82.6	20.1	93.3	26.2	86.6	18.2	89.0	19.3
EX06	100	65.1	63.0	10.6	11.2	83.8	20.1	93.7	26.1	86.4	18.2	86.7	19.3

**Table 3:** Thermal performance, measurement error, condensate amount, permeate flux, and membrane module pressure drop for MM001.

	Q_m_ (L/h)	Thermal Performance (kW)	Error (%)	Amount of Condensate (mL/h)	Permeate Flux (kg/m^2^h)	Pressure Drop of Membrane Module (kPa)
EX01	60	0.73	3	780	0.62	26
EX02	100	0.72	5	790	0.64	44
EX03	60	0.73	3	800	0.66	23
EX04	100	0.72	5	880	0.71	40
EX05	60	0.78	3	950	0.77	21
EX06	100	0.88	3	976	0.79	35

**Table 4:** Measured temperatures and humidity for combination of MM004 and MM007 and two condensers.

	Q_m_ (L/h)	T_mi_ (°C)	T_mo_ (°C)	T_ci_ (°C)	T_co_ (°C)	H1 (%)	T1 (°C)	H3 (%)	T3 (°C)	H4 (%)	T4 (°C)
EX07	100	55.4	53.3	11.5	11.8	68.6	21.6	86.2	17.1	75.9	19.4
EX08	150	55.2	53.6	11.4	11.8	68.6	21.4	86.2	17.6	76.0	19.8
EX09	120	59.0	57.3	11.3	11.7	68.5	21.13	87.0	17.2	75.9	19.4
EX10	150	61.4	59.9	11.4	11.8	70.6	21.35	85.9	17.5	76.9	19.5
EX11	360	65.5	64.9	11.6	12.0	70.4	21.4	87.6	17.7	77.7	19.7

**Table 5:** Thermal performance, measurement error, condensate amount, permeate flux, and membrane module pressure drop for combination of MM004 and MM007 and two condensers.

	Q_m_ (L/h)	Thermal Performance (kW)	Error (%)	Amount of Condensate (mL/h)	Permeate Flux (kg/m^2^h)	Pressure Drop of Membrane Modules (kPa)
EX07	100	0.85	3	185	0.46	3
EX08	150	1.0	4	240	0.60	5
EX09	120	0.89	4	220	0.55	4
EX10	150	0.96	2	260	0.65	5
EX11	360	0.95	4	300	0.75	21

**Table 6:** Measured temperatures and humidity for MM003.

	Q_m_ (L/h)	T_mi_ (°C)	T_mo_ (°C)	T_ci_ (°C)	T_co_ (°C)	H1 (%)	T1 (°C)	H3 (%)	T3 (°C)	H4 (%)	T4 (°C)
EX12	180	54.7	54.1	12.4	12.8	81.4	21.5	90.0	18.9	79.0	20.3
EX13	480	60.0	59.8	12.6	12.9	77.0	24.2	88.0	21.1	77.0	22.6
EX14	180	64.6	63.8	12.8	13.2	78.4	24.1	92.6	21.2	78.5	22.4

**Table 7:** Thermal performance, measurement error, condensate amount, permeate flux, and membrane module pressure drop for MM003.

	Q_m_ (L/h)	Thermal Performance (kW)	Error (%)	Amount of Condensate (mL/h)	Permeate Flux (kg/m^2^h)	Pressure Drop of Membrane Modules (kPa)
EX12	180	0.47	3	140	1.75	69
EX13	480	0.51	2	170	2.13	100
EX14	180	0.6	1	190	2.38	68

**Table 8:** Measured temperatures and humidity for MM006.

	Q_m_ (L/h)	T_mi_ (°C)	T_mo_ (°C)	T_ci_ (°C)	T_co_ (°C)	H1 (%)	T1 (°C)	H3 (%)	T3 (°C)	H4 (%)	T4 (°C)
EX15	240	54.3	53.7	11.4	11.8	75.7	21.7	90.0	18.9	79.0	20.3
EX16	300	59.1	58.5	11.1	11.6	80.8	19.7	88.0	21.1	77.0	22.6
EX17	300	64.4	63.9	11.1	11.6	84.0	20.0	92.6	21.2	78.5	22.4

**Table 9:** Thermal performance, measurement error, condensate amount, permeate flux, and membrane module pressure drop for MM006.

	Q_m_ (L/h)	Thermal Performance (kW)	Error (%)	Amount of Condensate (mL/h)	Permeate Flux (kg/m^2^h)	Pressure Drop of Membrane Modules (kPa)
EX15	240	0.6	1	180	1.13	35
EX16	300	0.76	1	190	1.19	40
EX17	300	0.76	3	220	1.38	40

**Table 10:** Measured temperatures and humidity for MM002.

	Q_m_ (L/h)	T_mi_ (°C)	T_mo_ (°C)	T_ci_ (°C)	T_co_ (°C)	H1 (%)	T1 (°C)	H3 (%)	T3 (°C)	H4 (%)	T4 (°C)
EX18	270	55.1	54.7	11.9	12.2	66.1	21.1	73.4	19.1	77.7	20.5
EX19	270	60.0	59.7	12.0	12.3	80.0	21.5	87.0	18.9	77.0	20.3
EX20	270	65.2	64.9	12.1	12.4	81.0	21.6	88.0	19.0	78.0	20.4

**Table 11:** Thermal performance, measurement error, condensate amount, permeate flux, and membrane module pressure drop for MM002.

	Q_m_ (L/h)	Thermal Performance (kW)	Error (%)	Amount of Condensate (mL/h)	Permeate Flux (kg/m^2^h)	Pressure Drop of Membrane Modules (kPa)
EX18	270	0.45	2	130	2.61	100
EX19	270	0.41	3	130	2.61	100
EX20	270	0.41	1	130	2.61	100

**Table 12:** Comparison of experiments EX14, EX17, and EX18 with other published results.

Reference	Solution (Concentration)	Feed Input Temperature (°C)	Permeate Flux (kg/m^2^h)	Flowrate (L/h)	Flow Velocity (m/s)	Amount of Condensate (mL/h)
EX14	water	65	2.38	180	1.24	190
EX17	water	65	1.38	300	1.38	220
EX18	water	55	2.61	270	0.62	130
Matheswaran et al. [60]	HNO_3_ (252 g/L)	60	0.5	3	n/a ^1^	n/a ^1^
Geng et al. [61]	NaCl (30 g/L)	70	2.3	10	n/a ^1^	n/a ^1^
Ho et al. [62]	NaCl (35 g/L)	55	4.659	54	n/a ^1^	n/a ^1^
Eykens et al. [63]	water	60	5	n/a ^1^	0.13	n/a ^1^

n/a ^1^—not available.

## Data Availability

The data presented in this study are available on request from the corresponding author.
